# A Framework for Detecting System Performance Anomalies Using Tracing Data Analysis

**DOI:** 10.3390/e23081011

**Published:** 2021-08-03

**Authors:** Iman Kohyarnejadfard, Daniel Aloise, Michel R. Dagenais, Mahsa Shakeri

**Affiliations:** Department of Computer and Software Engineering, Polytechnique Montreal, Montreal, QC H3T 1J4, Canada; michel.dagenais@polymtl.ca (M.R.D.); mahsa.shakeri@polymtl.ca (M.S.)

**Keywords:** anomaly detection, machine learning, performance evaluation, operating system, tracing

## Abstract

Advances in technology and computing power have led to the emergence of complex and large-scale software architectures in recent years. However, they are prone to performance anomalies due to various reasons, including software bugs, hardware failures, and resource contentions. Performance metrics represent the average load on the system and do not help discover the cause of the problem if abnormal behavior occurs during software execution. Consequently, system experts have to examine a massive amount of low-level tracing data to determine the cause of a performance issue. In this work, we propose an anomaly detection framework that reduces troubleshooting time, besides guiding developers to discover performance problems by highlighting anomalous parts in trace data. Our framework works by collecting streams of system calls during the execution of a process using the Linux Trace Toolkit Next Generation(LTTng), sending them to a machine learning module that reveals anomalous subsequences of system calls based on their execution times and frequency. Extensive experiments on real datasets from two different applications (e.g., MySQL and Chrome), for varying scenarios in terms of available labeled data, demonstrate the effectiveness of our approach to distinguish normal sequences from abnormal ones.

## 1. Introduction

In recent years, computing infrastructure has significantly evolved, whereas complex systems have facilitated many complicated and large-scale tasks. For example, functional co-processing units accommodate conventional processing units to speed up particular tasks such as virtualization or complex machine learning computations. Consequently, a simple operation can involve multiple parallel cores, being served in a few seconds or milliseconds. These improvements have increased the expectation level of the users, so that any performance fluctuations or increased latency may lead to user dissatisfaction and financial loss. Different reasons such as software bugs, misconfigurations, network disconnection, hardware faults, aging phenomena of the systems, or even extreme load injected by other applications into the system, may degrade the performance of a particular service or application. Hence, monitoring and analyzing the performance of applications to find any performance anomaly or degradation is of particular importance. Indeed, any delay in detecting performance problems and troubleshooting can significantly increase the cost to fix them.

Performance anomaly detection refers to the problem of finding exceptional patterns in execution flow that do not conform to the expected normal behavior. Many sources may cause performance anomalies, such as application bugs, updates, software aging phenomenon, and hardware failure. It should be noted that performance anomalies are different from high resource consumption. An application might be inherently CPU or I/O intensive without being categorized as anomalous. However, imposing a continuous and more than expected average workload intensity on the system can be a sign of an anomaly. Relying on the definition of performance anomaly detection, we believe that whatever the source of the anomaly is, it makes the execution’s flow different from the normal situation. Consequently, it seems interesting to look at the problem from a more general point of view and try to find the deviations of the execution’s flow, regardless of the source of the anomaly. In case of any abnormal behavior during software execution, system developers or experts need information that not only locates that behavior but also provides details of the execution at the time the anomaly occurs. The performance metrics provided by tools such as top, etc., can represent the average load on the system. However, they do not help detect anomalies since a live threshold subject to the current system state would be needed to distinguish whether the application’s behavior is normal or abnormal, which is practically impossible. Even if such thresholds were available, these tools would not provide any details about the application’s execution flow. Therefore, system experts often employ logs and low-level tracing tools to define efficient strategies to find anomalies as well as their causes. Tracing is an effective way of gaining information on a system for further analysis and debugging of the underlying system, while minimizing monitoring influence [1]. However, it is an exhausting responsibility for human administrators to manually examine a massive amount of low-level tracing data and monitor the execution status of an application [2]. Hence, an accurate anomaly detection framework with minimum human intervention is in order.

Tracing data can offer detailed information about the execution of applications and processes. System calls are essential traceable events that contain valuable information about the program’s flow. They represent low-level interactions between a process and the kernel in the system. Processes must interact with the operating system for each request, such as opening a file, writing into the registry, or opening a network connection, which is done through system calls. A system call trace provides an ordered sequence of system calls that a process performs during its execution. The definition of normal behavior is stable for standard UNIX process [3]. When a process is anomalous, its system call trace is extremely different from the process running under normal conditions [4,5]. Our goal in anomaly detection is to find sets of system calls that are not likely to happen together in normal situations.

This work proposes a general anomaly detection framework to process the large volume of tracing data by taking advantage of machine learning technologies and open-source tools (i.e., LTTng and Trace Compass). Its main contributions are the following: **First**, unlike many other methods that use performance metrics or unstructured logs, we employed LTTng for data collection, which provides a system software package for correlated tracing of the Linux kernel, applications, and libraries [6]. LTTng provides high-resolution details of the program’s execution by presenting kernel and userspace events related to the moment anomalies occur.  **Second**, this article has addressed the problem of availability of labeled data by proposing learning techniques depending on their volume. Consequently, when a large amount of labeled training data is available, a supervised method is introduced, whereas an unsupervised method is preferred when labeled data is not available. Moreover, we propose a novel semi-supervised machine learning model within proposed framework that benefits from both supervised and unsupervised learning techniques when only a few labeled data are available. It should be noted that all proposed learning methods use the same data structure. **Third**, this is the first time that the durations of the most important system calls are used to make feature vectors. The duration of a system call in a window acts like the weighted frequency of that system call. Further, using the most important system calls instead of the whole set of system calls is novel, and it is shown to improve detection performance. **Fourth**, the proposed anomaly detection framework reduces troubleshooting time and directs the developer or troubleshooter to discover the problem by highlighting the anomalous parts of the trace. It helps developers look at just a few small windows instead of the whole trace includes millions of events. Using the proposed anomaly detection framework alongside the Trace Compass gives the developers a deep understanding of what happened at the time of the anomaly. It enables developers to use many preexisting scripts and views in Trace Compass for further analyzing the anomaly detection output.

The rest of the paper is organized as follows. In Section 2, related studies are presented. In Section 3, we describe the details of the performance anomalies in processes. In Section 4, we introduce our automatic integrated anomaly detection framework. Section 5 discusses the algorithm for kernel tracing and data extraction. Preprocessing of the extracted data is explained in Section 6. Then, the feature selection strategy along with supervised, unsupervised, and semi-supervised anomaly detection methods are proposed in Section 7. Section 8 provides the experimental results from two different applications (i.e., MySQL and Chrome), followed by the conclusions in Section 9.

## 2. Previous Work

In this section, the available techniques for performance anomaly detection are reviewed. The earliest efforts consisted of statistical methods [7]. These works keep the activity of subjects and generates profiles to represent their behavior. Profiles include measures such as activity intensity measure, audit record distribution measure, categorical measures, and ordinal measure. As events are processed, an anomaly score is computed using an abnormality function and profiles. If the anomaly score is higher than a certain threshold, the detection system generates an alert. Statistical models have some disadvantages. Defining proper thresholds which can balance the likelihood of false positives and false negatives is very difficult to set. Moreover, most of the statistical anomaly detection techniques require the assumption of a quasi-stationary process. However, this cannot be assumed for most data processed by anomaly detection systems. Wang and Battiti [8] proposed a method in which the distance between a vector and its reconstruction onto the reduced PCA subspace represents whether the vector is normal or abnormal. This method is limited to pre-determined anomalies and is not able to detect novel types of anomalies, besides suffering from the problem of defining thresholds.

In addition to these methods, several machine learning-based schemes have been applied to detect anomalies in systems. They work based on the establishment of a model that allows the patterns to be categorized [9]. Bayesian networks can encode probabilistic relationships among variables of interest, thereby predicting the consequences of an event in the system [10]. Ye and Borror presented a cyber-attack detection technique through anomaly detection using a Markov chain [11]. Achieving high performance in these techniques depends on the quality of the data. This is because the Markov Chain technique is not robust to outliers and performs better when the amount of noise in data is low [12]. Besides, these models have better performance for small datasets. Among other approaches, clustering algorithms can detect abnormal behavior without prior knowledge. Many clustering algorithms, such as *k*-means, *k*-medoids, EM Clustering, and outlier detection algorithms, have been employed for anomaly detection. In  [13], the *k*-Means clustering algorithm with the accompaniment of different dimensionality reduction modules (PCA, ICA, GA, and PSO) was used to separate time intervals of the traffic data into normal and anomalous groups. However, none of these works have mentioned how to collect the data. These works are limited to clustering preexisting datasets and do not provide a solution for real-world usage. Apart from clustering methods, classification-based anomaly detection approaches like support vectors, Fuzzy Logic, and Neural Networks have been widely used in this area [14]. In  [15], a fuzzy technique is proposed to extract abnormal patterns based on various statistical metrics in which fuzzy logic rules are applied to classify data [15]. Statistical metrics cannot be used to find the root cause of the anomaly after detecting an anomaly because these metrics do not provide details of the execution flow.

One imperative point in system performance analysis is how to characterize the executing software. In this regard, behavioral analysis techniques can be used to automatically monitor the performance of the processes running on a system. Some other studies have used system calls to characterize software behavior. Forrest et al. [3] showed that during the normal execution of a program, a consistent sequence of system calls is generated. In their method, all possible normal patterns of different lengths are collected to form the normal dataset. Then different patterns in the new trace are compared with the normal dataset, and any deviation from the normal model is considered an anomaly. The first weakness of this method is that finding all the patterns with different lengths is extremely time-consuming because a short tracing file includes thousands of events. Furthermore, the resulting database is massive. It is notably time-consuming to compare a new pattern to the entire normal dataset.

The use of system calls has led to a dramatic improvement in anomaly detection techniques. Canzanese et al. characterized system call traces using a bag-of-n-grams model, which represents a system call trace as a vector of system call n-gram frequencies [16]. In this regard, Kolosnjaji et al. [17] attempted to apply deep learning to model the malware system call sequences for malware classification. They constructed a neural network based on convolutions in order to obtain the most desirable features of n-grams. A well-known issue with N-gram-based approaches is sparsity. The N-gram model, like many statistical models, is significantly dependent on the training data. Besides, the performance of the N-gram model varies with the change in the value of N. In [18], Strace is utilized for collecting logs, and then the Linux kernel system calls are extracted to construct weighted directed graphs. This method in which the graph-based representation is used for anomaly detection suffers from the high cost of obtaining such graphs. Finding related system calls out of thousands of events requires extremely high computational power.

Many sources may cause anomalies or performance degradation, such as application bugs, updates, software aging phenomenon, and hardware failure. Various articles have tried to discover or solve performance degradations resulting from each of these sources. For example, software rejuvenation was introduced to prevent or at least delay aging-related failures [19]. Software aging has been demonstrated to affect many long-running systems, such as web servers, operating systems, and cloud applications. Ficco et al. have examined the effects of software aging on the gradual increase in the failure rate or performance degradation of Apache Storm over time [20]. Apache Storm is an open-source distributed real-time computational system for processing data streams. These systems may be affected by software aging because they usually run for a very long time. In their work, the measures related to the system resources usage and the user-perceived performance are collected by vmstat utility and by reading from Storm logs details about emitted requests and their responses. This information is employed to discover evidence of software aging. However, software aging is just one of several sources of anomalies. Relying on the definition of anomaly, we believe that whatever the source of the anomaly is, it makes the execution’s flow different from the normal situation. Hence, it seems interesting to look at the problem from a more general point of view and try to find the deviations of the execution’s flow, regardless of the source of the anomaly. In addition, LTTng can gather kernel events as well as the userspace events without imposing much overhead to the system. LTTng has several features that make it usable for most Linux-based environments. For instance, the LTTng relay daemon enables us to trace distributed systems.

Our work distinguishes from the previous related literature since:

- Unlike most previous works, which did not provide a solution for data collection, we defined our data collection module using LTTng and Trace Compass. Using various LTTng features makes our proposed framework applicable in most Linux-based environments without much change. For instance, the LTTng relay daemon enables us to trace distributed systems and cloud environments. In addition, no special settings are employed while collecting the data. We used tracing in our proposed framework because tracing enables us to examine the execution flow using tools such as Trace Compass.
- Statistical metrics can not be used to find the cause of the anomaly after detecting an anomaly. Compared to statistical techniques, our proposed framework has no assumption and is not dependent on the existence of any threshold. This fact and the way we use the system calls increase the generality of our method and make it usable for any application and environment.
- Achieving high performance using Bayesian networks and Markov chain techniques depends on the quality of the data. These techniques are not robust to outliers and perform better when the amount of noise in data is low. Besides, these models have better performance for small datasets. These problems were solved in our work by carefully choosing the learning method so that the presence of noise or new data points does not cause much change in the model and works appropriately for large data.
- Many of the available performance anomaly detection approaches use supervised methods, which require labeled data. However, labeled data is not always available. While proposing an unsupervised approach is desirable, it is a great challenge to achieve high accuracy by means of an unsupervised method. We have provided a package of supervised, unsupervised, and semi-supervised methods that can be used according to the volume of available labeled data. All these three methods use the same data structure, and no special settings are employed while collecting the data. After training the model in our proposed method, the detection is done very quickly and without high computational cost.
- Unlike methods that compare a pattern to all normal patterns in a database to determine if it is abnormal, our method is not limited to a primary database. Finally, presenting events that occurred during the anomaly helps the developer not spend much time examining the entire events in a trace or log file in order to discover the anomaly’s cause.

## 3. Performance Anomaly in Processes

Performance anomalies are the most significant obstacles to the system to perform confidently and predictably in enterprise applications. Many sources can cause anomalies, such as varying application load, application bugs, updates, and hardware failure. In a situation where the workload is the source of the anomaly, the application imposes continuous and more than expected average workload intensity to the system. Faults in system resources and components may considerably affect application performance at a high cost [21]. In addition, software bugs, operator errors, hardware faults, and security violations may cause system failures.

The preliminary performance profiling of a process that reflects its typical behavior can be done using synthetic workloads or benchmarks. At a higher level, the performance of computer systems is delineated by measuring the duration of performing a given set of tasks or the amount of system resources consumed within a time interval [22].

There exist many metrics for measuring the performance of a system. Latency and throughput are the most used ones. They are used to describe the operation state of a computer system. The time that passes between the beginning of an operation and its completion is the latency, (e.g., the delay between when a user clicks to open a webpage and when the browser displays that webpage). Throughput is a measure of how many jobs a system can perform in a given amount of time (e.g., the number of users’ requests completed within a time interval). In addition, resource utilization of an application indicates the amount of resources (e.g., number of CPUs, and the size of physical memory or disk) used by that application. The CPU utilization is the percentage of time in which the CPU is executing a process whereas the memory utilization is the amount of storage capacity dedicated to a particular process.

**Figure 1:**
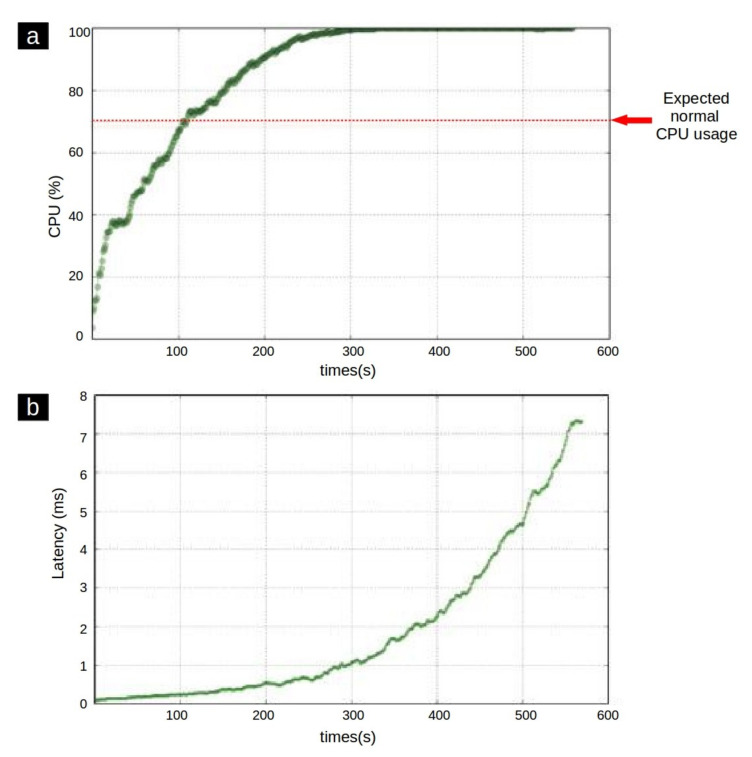
(**a**) CPU usage of an application during the time. (**b**) an anomalous latency growth pattern [24].

Figure 1a shows an example of the CPU utilization of a process during its lifetime. When an application is running normally, the CPU used by that application is conventional. Hence an expected maximum CPU utilization threshold can be defined for each application. In this case, if the CPU usage exceeds the threshold value, the process behavior is prone to the existence of an anomaly. Furthermore, as represented in Figure 1b, during the anomalous running of a process, the latency is usually increased while this curve has a relatively steady trend during normal behavior [23].

From another perspective, data anomalies can be defined in various forms. Two principal forms of anomalies are point anomalies and collective anomalies. Point anomalies are data points that are different from normal data. For example, consider a situation where data is generated from different data distributions, each one defining a cluster. In this case, data points which do not seem to have been generated by the data distributions are considered as point anomalies. While searching this type of anomalies, performance metrics such as CPU utilization or throughput can be used to determine if abnormal behavior has occurred at a particular timestamp. In the case of collective anomalies, we cannot detect individual data points as anomalies by themselves; however, their collective occurrence together may be an anomalous behavior. In this method, instead of detecting an anomaly at a particular timestamp, the system’s behavior during a sequence of events is investigated. Due to the use of tracing data and the fact that a single event obtained from tracing does not contain enough information to detect an anomaly, we search mostly for collective anomalies in this work. Besides, looking at the sequence of events provides insightful information about the system behaviors over a period of time, which is essential for analyzing the root cause of an anomaly. Finally, by targeting the collective anomalies, our framework can even handle rare system call invocation paths. For example, a user never opens FTP connections on Chrome, but one day decides to do so. This will lead to a significantly different invocation path. In practice, observing a new system call is not a reason for an anomaly to occur, and we cannot consider a subsequence of events to be abnormal only due to the presence of a new system call. The subsequence in which the rare system call occurs is considered abnormal not only because of that system call but also because of the effects the system call has on the surrounding system calls. This system call may also be ignored during the feature selection process, in which case its effect is still present in the subsequence.

Anomalies can be defined from the user experience aspect, and in many situations, anomalies happen on the server-side, but their effect can be realized on the user side. Moreover, a physical or virtual node is often not dedicated to a unique particular service. So, latency or throughput in a sampling period cannot help to find anomalies in a program execution while several programs are running on the node. In this case, separating the normal and the abnormal behavior is very difficult, and the result depends on the hardware. Furthermore, the latency or throughput does not contain execution details, while the sequence of events such as system calls reveals many details about program execution.

## 4. The Automatic Integrated Anomaly Detection Framework

**Figure 2:**
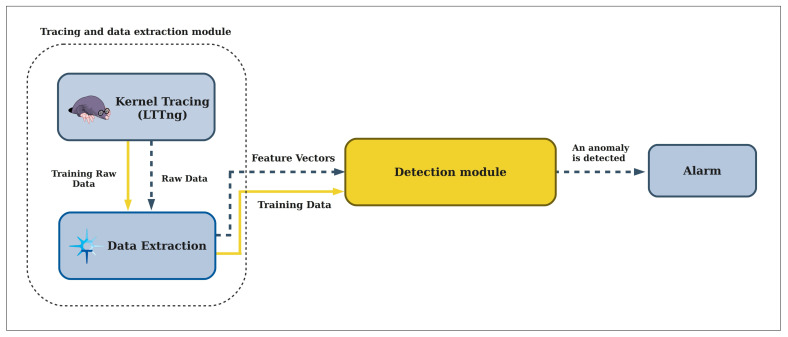
The system architecture of the proposed framework.

In this work, we propose an automatic anomaly detection framework to process the large volume of tracing data by taking advantage of machine learning technologies. The system architecture of the proposed framework is shown in Figure 2. The generality of the framework is extremely important, and it must be capable of working along with any program or system with different settings.

As illustrated in Figure 2, the entire framework is divided into several modules. First, kernel tracing is done to gather the system calls information during the execution of a program. We employ LTTng (Linux Trace Toolkit Next Generation) in this module, a system software package for correlated tracing of the Linux kernel, applications, and libraries. The raw tracing data is fed into the data extraction module that processes it with a windowing method that will be introduced later in this paper. Data transformation and feature extraction is done in the Trace Compass application. This module is responsible for preparing data for the detection module in both the training and detection phases. This data contains feature vectors extracted from the tracing data. When the model is trained, the data extraction module sends new feature vectors to the detection module at detection time. We discuss each module in the following sections.

Our anomaly detection framework’s design aims to provide high accuracy and time efficiency in analyzing tracing data and detect anomalous performance behaviors in large scale systems. By investigating the required framework specifications it seems challenging to apply an anomaly detection framework in practice because of two issues. The first is that continuously collecting system calls for machine learning methods is computationally expensive and of rapid storage increase. Furthermore, the machine learning model itself takes a long time to train. To address the latter issue, we assume that once the model has been trained for an application, there is no need to retrain it, and periodic updates are enough. However, continuously collecting system calls is still needed. Hence, we propose using LTTng-rotate for increasing data collection efficiency, thus reducing the size of the tracing file.

## 5. Kernel Tracing and Data Extraction

In this section, the data extraction technique is explained in which two data sets are created by tracing the underlying system kernel by means of a sliding windowing technique. We use our data collection instead of using many existing systems calls data sets. Our data extraction technique allows us to collect our own fields (name, index, and especially duration) and trace any program. Furthermore, we can consider the time of data collection in the overall process because the data collection is not free as it is considered in many existing works, and finally, the collaborations required to handle a request can be considered, which is not the case in the existing data sets.

Tracing is a popular technique to analyze, debug, and monitor processes running on a system. Furthermore, it is an efficient way of gaining information on a system while minimizing the monitoring influence. The instrumentation of the traced application provides as output timestamp-matched events and intuition on the execution of various parts of a system. Thus, the precision of the monitored events is equal to the internal clock of the device.

**Figure 3:**
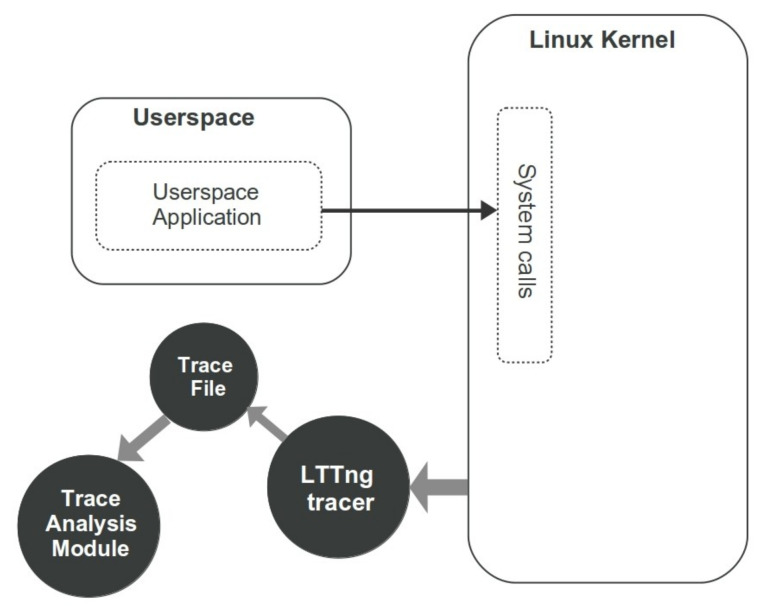
Data extraction steps using kernel tracing.

Traces are massive data which can be fed into a machine learning framework. Fortunately, several standard tools and tracing methodologies exist in different environments. Here, in order to analyze the behavior of each process and find out the performance status, each system is equipped with a lightweight tracing tool called the Linux Trace Toolkit Next Generation(LTTng) [6]. It is implemented for high throughput tracing and includes multiple modules for Linux kernel and userspace tracing with minimal cost. Tracing the OS or user applications requires the ability to record thousands of low-level events per second which imposes some overhead to the system that may affect the performance of the target application. Hence, LTTng is a proper tool to be used in our experiment as we would like a tracer to have a low overhead on the monitored systems. Figure 3 represents the process of collecting kernel events in a trace file and transferring it into the Trace Analysis module. As illustrated in this figure, the userspace application sends requests to the Linux kernel using system calls which are recorded by LTTng Tracer on .ctf trace files. In the sequel, the trace file is fed into the trace analysis module to create the dataset and perform more investigation.

**Figure 4:**
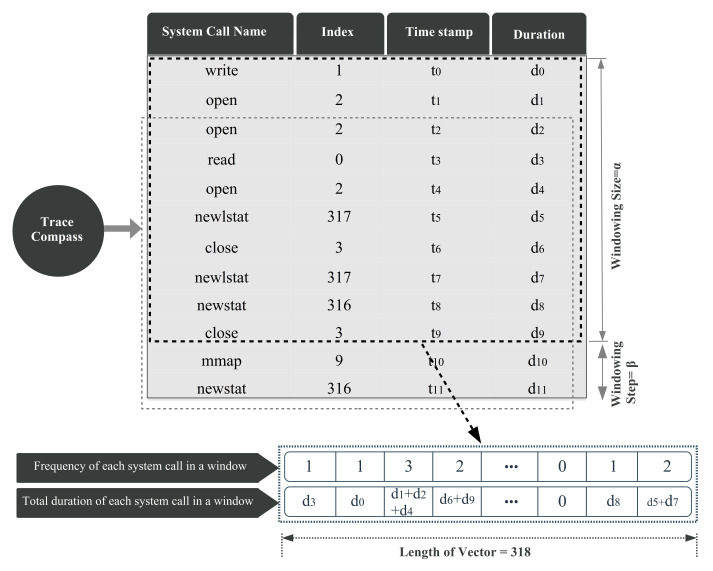
Reading trace file and extracting vectors using windowing method.

We implemented the trace analyzer module within the Trace Compass open source tool [25], with visualization mechanisms to promote the analysis of the system performance anomalies with different perspectives. Actually, the LTTng tool is applied to collect system calls originated by the monitored processes, and Trace Compass is employed to read the LTTng trace files and to produce a sequence of events with all their associated information (e.g., system call name, timestamp, and duration). Our methodology focuses on system calls, so while the Trace Compass code is reading the trace file, it only collects system calls and skips other events. In the obtained dictionary, each system call entry contains a timestamp, process ID, and some additional run-time information associated with that system call, which is depicted in Figure 4.

First, the processes other than the one under study (e.g., MySQL and Chrome) are filtered out considering the process ID field. Then, instead of working with system call names, an index is assigned to each system call. The system calls indices, the corresponding execution times, and other related information are listed for all threads of the target process. Since a single process can produce a huge amount of system calls, considering all system calls at once is not practical in real applications. Therefore, a sliding window is used to continuously extract data from subsequences of system calls. For each subsequence, we define a compact representation that yields two separate feature vectors containing the frequency ($x_{frequency}$) and the duration ($x_{duration}$) of the system calls inside the current sliding window. Thus, our methodology can handle large and varying volumes of data. Since we monitor 318 different system calls of the Linux operating system, each feature vector has 318 dimensions, one per system call type. This feature extraction strategy is shown in Figure 4.

The pseudocode for extracting the feature vectors is represented in Algorithm 1. The algorithm receives the windowing size α, the windowing step β, a Trace τ which contains a sequence of events, and the target process *m* as input. Some factors must be considered when selecting α and β values. Windows must contain sufficient information about the status of the system over a period of time. In one hand, choosing a small amount as the length of the window reduces the useful information volume of the subsequence and increases the number of subsequences. Furthermore, it may even increase sparsity. In the other hand, if a large α value is selected, it is likely that the screened subsequences contain both normal and abnormal events. Moreover, small β values make the subsequences very similar, and larger values also ignore many possible subsequences. There is no need to worry about calculating these values. It can be a manual trial-and-error process to performed at training-validation time. It does not impose much computation cost to the whole framework.
**Algorithm 1** Feature extraction procedure.**Input:** Trace τ, Process *m*, α, β**Output:** *D*, *F*1:D←∅, F←∅2:$SP=\left\{e\in \tau |\;type(e)=systemcall\;and\;process(e)=m\right\}$3:*W*← MakeSubsequences(SP, α, β)4:**for** all $i\in \left\{1,2,...,|W|\right\}$ **do**5:$FV\leftarrow \sum_{j=1}^{\alpha }R_{i,j}$6:$DV\leftarrow \sum_{j=1}^{\alpha }S_{i,j}$7:$F\leftarrow F\cup \left(FV\right)$8:$D\leftarrow D\cup \left(DV\right)$9:**end for**

Algorithm 1 first obtains the set of frequency based feature vectors (*F*) and the set of duration based feature vectors (*D*). At the beginning of the algorithm, the system calls belonging to the process *m* are extracted from the total events in the trace file and a set SP is built (line 2). The function type(e) determines if the event *e* is a system call or not. Then in line 3 the function MakeSubsequences() obtains all possible subsequences in SP by considering the widowing size α and the windowing step β. For each subsequence $W_{i}$ two data structures of size (α×318) are built: $R_{i}$ and $S_{i}$. Let $R_{i,j}$ be a (1×318) one-hot vector which corresponds to the *j*-th system call done in the *i*-th subsequence. In this vector, the *k*-th cell where *k*=index($w_{i,j}$) is equal to one. The vector FV is calculated by the sum of all the one-hot vectors $R_{i,j}$ for all $j\in \left\{1,2,...,\alpha \right\}$. In a similar way, $S_{i,j}$ represents a (1×318) one-hot vector which corresponds to the *j*-th system call done in the *i*-th subsequence.. In this vector, all the cells have zero value except the *k*-th cell where *k*=index($w_{i,j}$). The value of this cell is equal to the duration of that system call. The vector DV is computed by the sum of all the one-hot vectors $S_{i,j}$ for all $j\in \left\{1,2,...,\alpha \right\}$. Finally, *D* and *F* provide a set of duration vectors and a set of frequency vectors, correspondingly (lines 7 and 8).

## 6. Preprocessing of the Extracted Data

Data preprocessing is an essential stage for the success of any machine learning model. In almost all knowledge discovery tasks, the data preprocessing step takes the major part of the overall development effort, even more than the data mining task [26].

### 6.1. Problem of Sparsity

Each subsequence of events present in a window is represented by a frequency (duration) vector with the size of total number of system calls (i.e., 318). Naturally, most of the values in each vector will be zero due to the large number of system calls. Besides, a specific process utilizes special system calls during its execution. In other words, some columns of the data sample will consist of zero values. This characteristic dramatically impacts calculating sample similarities. Moreover, it is hard to understand the relationships between different feature vectors when the training set is not large enough in the presence of sparsity [27]. Thus, in this paper, we reduce the sparsity of the collected data by eliminating all unused features related to system calls that never occur during the execution of the monitored process.

### 6.2. Data Normalization

Data normalization is a fundamental phase of data preprocessing. Data normalization is employed to reduce the dominating effect of some attributes measured in different scales. Here, data standardization is applied on the dataset as a normalization preprocessing step. Let, $\Gamma =\left\{X_{1},X_{2},...,X_{n}\right\}$ denote the d-dimensional data set. Thus, Γ is a n×d matrix:

(1) $$\begin{array}{c} \Gamma =\left[\begin{array}{ccc} x_{11} & ... & x_{1d}\\ ... & ... & ...\\ x_{n1} & ... & x_{nd} \end{array}\right] \end{array}$$

Given a dataset Γ, the Z-score standardization formula is defined as:

(2) $$\begin{array}{c} x_{ij}=Z\left(x_{ij}\right)=\frac{x_{ij}-\mu_{j}}{\sigma_{j}}, \end{array}$$

where $\mu_{j}$ and $\sigma_{j}$ are, respectively, the samples mean and the standard deviation of the *j*th attribute. This method rescales the features in a way that they have a standard normal distribution with mean of 0 and standard deviation of 1.

## 7. Performance Anomaly Detection

In this section, first, we assume that enough labeled training samples are available. Thus, we propose a supervised monitoring framework that classifies the system performance into three separated classes: normal, CPU issue, and Memory issue. Although a supervised approach could usually produce acceptable detection results, it requires enough labeled data. Since providing labeled data for the whole data distribution is not always possible, we propose to use an unsupervised approach in Section 7.2. The unsupervised approach does not require any labeled data and clusters the input data into separate categories, which could represent different groups of normal, CPU issue, or Memory issue. However, unsupervised approaches usually present worse classification performance than supervised methods in practice given that no priori information is exploited. Therefore, in order to introduce a from of supervision into the unsupervised approach and improve the detection performance, we propose a semi-supervised approach in Section 7.3. In this method, we assume that a subset of data is labeled and can be used to guide the feature selection procedure. In this way, the benefits of the supervised and unsupervised learning strategy are combined into a semi-supervised anomaly detection approach.

### 7.1. Supervised Performance Anomaly Detection

Once the system call feature vectors are collected per subsequence, the purpose of the anomaly detection algorithm becomes to train a model with normal and abnormal data from the provided labeled training dataset. Later, the task would be to determine whether a test sample vector belongs to a normal or abnormal behavior. Here, we describe a supervised monitoring framework that classifies the system performance into three separate classes. If a vector has a normal behavior, it will be assigned to the first category. The second class is defined as a CPU issue or, in other words, insufficient CPU allocation problem, which may happen when the system is running a CPU intensive process. Finally, the vectors extracted from a system running a memory-intensive process are assigned to the third category. This class indicates an insufficient memory allocation issue.

#### 7.1.1. Iterative Feature Selection

Feature selection is the process of finding the most discriminative subset of features for performing classification. In the case of supervised learning, this selection is performed based on the available labeled data. A proper feature selection can improve the learning accuracy and reduce the learning time. Here, the Fisher score along with a correlation filtering strategy [28] are applied to determine the best subset of features in the dataset. In this algorithm, a subset of features are found in a way that the distances between samples in different classes become as large as possible, while the distances between data points in the same class stay as small as possible. The Fisher Score $FS_{j}$, for j=1,…,d, can be calculated as follows:

(3) $$\begin{array}{c} FS_{j}=\frac{\sum_{c=1}^{k}n_{c}{\left(\mu_{jc}-\mu_{j}\right)}^{2}}{\sum_{c=1}^{k}n_{c}\sigma_{jc}^{2}}, \end{array}$$

where $n_{c}$ is the number of samples in class *c*, for c=1,…,k (number of classes), and $\mu_{jc}$ corresponds to the average value of feature *j* restricted to the samples in class *c*. Further, $\sigma_{jc}^{2}$ is the variance of feature *j* for samples in class *c*.

The computed Fisher scores of each feature are sorted in non-increasing order and scanned iteratively to select the *ℓ* features that have low correlation together. A feature is selected to compose the list of *ℓ* features if its pairwise correlation with one of the features already selected in superior to a given threshold. This procedure continues iteratively until *ℓ* features are selected. Here, the correlation between two features $j_{1}$ and $j_{2}$ is computed as follows:

(4) $$\begin{array}{c} Cov\left(j_{1},j_{2}\right)=\frac{\sum_{i}^{n}\left(x_{ij_{1}}-\mu_{j_{1}}\right)\left(x_{ij_{2}}-\mu_{j_{2}}\right)}{n-1} \end{array}$$

#### 7.1.2. Supervised Multi-Class Anomaly Detection

Once the top-ranked features are selected, we employ a multi-class support vector machine (SVM) [29] classification model. We choose SVM considering its generalization ability and its successful utilization in different pattern recognition applications, such as anomaly detection tasks [30]. SVM finds the hyperplane with the largest margin that classifies the training set samples into two classes. Then the unseen test samples are labeled by checking the sign of the hyperplane’s function.

Considering each sample $X_{i}$, for i=1,…,n of the training data and its associated label $y_{i}$, SVM finds the optimal hyperplan by solving the following problem:

(5) $$\begin{array}{c} \min_{\omega ,d}\frac{1}{2}\omega^{T}\omega +C\sum_{i=1}^{n}\xi_{i} \end{array}$$

(6) $$\begin{array}{c} s.t.\;y_{i}\left(\omega^{T}\phi \left(X_{i}\right)+c\right)\geq 1-\xi_{i}\;,\;\xi_{i}\geq 0\;,\;i=1,...,n \end{array}$$

where ω is d-dimensional vector and $\xi_{i}$ is a measure of the distance between the misclassified point and the separating hyperplane. The function $\phi \left(x_{i}\right)$ projects the original data sample $x_{i}$ into a higher dimensional space and *d* is the bias. *C* controls the penalty associated with the training samples that lie on the wrong side of the decision boundary. The radial basis function (RBF) of $\phi \left(x\right)=e^{{\gamma ∥\left(x-x_{i}\right)∥}^{2}}$ is applied to map the data into the non-linear high-dimensional space. The term γ is a parameter that controls the width of the Gaussian kernel. The accuracy of the classification is then dependent on the value of the parameters *C* and γ.

In this work, we generalize the binary classification model by means of a one-versus-one approach. In this approach, one classifier per pair of classes is built. In our case, it fits three classifiers for three possible pairs of classes: (1) samples with memory issues from the samples with CPU issues, (2) samples with memory issues from the normal samples, (3) samples with CPU issues from the normal samples. The class which received the most votes is selected at prediction time. In the case that two classes have an equal number of votes, it selects the class with the highest aggregate classification confidence by summing over the pairwise classification confidence levels computed by the underlying binary classifiers.

### 7.2. Unsupervised Learning of the Performance Anomalies

Most current anomaly detection systems use labeled training data. As mentioned before, producing this kind of training data is usually expensive. Besides, the definition of normal and anomalous behaviours may change over time. To address these problems, we propose to use an unsupervised system call based anomaly detection scheme. This technique segments unlabelled data vectors into distinct clusters. The proposed unsupervised approach should be able to categorize previously unseen types of anomalies. A wide variety of models for cluster analysis exists; however, the initial choices are usually representative-based algorithms such as K-Means, which directly uses the distances between the data points to cluster a dataset. Another clustering approach based on data density used in this work is DBSCAN which can group clusters of varied complex shapes. In the following, we briefly describe the *K*-Means and the DBSCAN algorithms.

#### 7.2.1. K-Means Clustering

K-Means is a clustering algorithm that groups samples based on their feature values into *k* different clusters. Data samples which are assigned to the same cluster are supposed to have similar feature values. In this clustering technique, the sum of the squares of the Euclidean distances of data points to their closest representatives is used as an objective function [31,32]:

(7) $$\begin{array}{c} Dist\left(X_{i},X_{j}\right)={∥X_{i}-X_{j}∥}_{2}^{2} \end{array}$$

where $X_{i}=\left(x_{i1},...,x_{id}\right)$ and $X_{j}=\left(x_{j1},...,x_{jd}\right)$ are two input vectors with *d* features and ${∥\cdot ∥}_{p}$ represents the $L_{p}-norm$. K-Means begins by initializing the *k* centroids using a straightforward heuristic like random sampling from the dataset and then refines the centroids in the following steps until stability is reached:

- Assign each vector to the closest centroid using the similarity function (Equation (7))
- Determine the optimal centroid for each cluster $C_{j}$

#### 7.2.2. Dbscan Clustering

**Figure 5:**
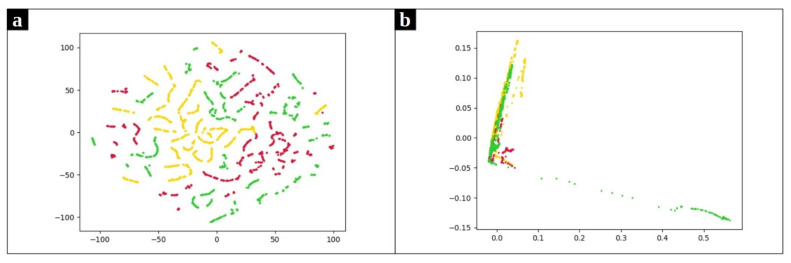
Frequency-based samples extracted from Chrome process. Red, yellow and green points refer to normal, CPU problems, and memory problems, respectively. (**a**) uses t-SNE and (**b**) utilizes PCA to map data points onto 2D subspaces.

The use of K-Means clustering has some limitations. First, it requires the user to set the number of clusters a priori. Second, the presence of outliers has an undeniable impact on *K*-means. Besides, *K*-means works better for spherical clusters considering the Euclidean space as the underlying data space. To further reveal this point, consider the clusters represented in Figure 5. These plots depict the frequency-based vectors extracted from a chrome process use case along with their real labels. Since the original data has more than 120 attributes, two separate dimensionality reduction approaches were applied to better visualize the data. In Figure 5a we present data obtained with the t-distributed Stochastic Neighbor Embedding (t-SNE) [33] while Figure 5b presents the data projected in the plane by means of PCA [34]. Both figures reveal that the *K*-means algorithm is not appropriate to correctly cluster the illustrated dataset. Here there are three clusters of arbitrary shape in the data, and thus density-based algorithms are preferable.

Hence, our proposal uses the DBSCAN algorithm [35], in which the individual data points in dense regions are used as building blocks after grouping them according to their density.

DBSCAN algorithm requires two parameters. The first parameter is ϵ, which defines the neighborhood around a data point. Two points are considered as neighbors if the distance between them is lower or equal to ϵ. If the ϵ value is chosen too small, then a large part of the data will be considered as outliers. On the other hand, if it is chosen very large then the clusters will merge, and the majority of the data points will be in the same cluster. The second parameter is MinPts, which indicates the minimum number of neighbors (data points) within ϵ radius. The density of a point is the number of points that lie within a radius ϵ of that point which can be obtained by the following formula:

(8) $$\begin{array}{c} N_{\epsilon }\left(X_{i}\right)=\left\{X_{j}\in Dataset|Dist\left(X_{i},X_{j}\right)\leq \epsilon \right\} \end{array}$$

DBSCAN classifies the data points into three categories of core, border, and outliers based on the hyperparameters ϵ and MinPts. A point is a core one if it has more than MinPts points within ϵ, and a border point is a point that has fewer than MinPts within ϵ, but it is in the neighborhood of a core point. A point that is not a core point or border point is considered as an outlier. Also, three terms required for understanding the DBSCAN algorithm: (1) point *A* is “directly density reachable” from point *B* if *A* is within distance ϵ from core point *B*. (2) A point *A* is “density reachable” from *B* if there is a set of core points leading from *B* to *A*. (3) Two points *A* and *B* are “density connected” if there is a core point *C*, such that both *A* and *B* are density reachable from *C*. A density-based cluster is defined as a group of density connected points. By considering these definitions, DBSCAN algorithm can be described in the following steps:

- For each point $x_{i}$, compute the distance between $x_{i}$ and the other points. Finds all neighbor points within distance ϵ of the starting point $x_{i}$. Each point, with a neighbor count greater than or equal to MinPts, is marked as core point or visited.
- For each core point, if it is not already assigned to a cluster, create a new cluster. Find all its density connected points recursively and assign them to the same cluster as the core point.
- Iterate through the remaining unvisited points in the dataset.

Those points that do not belong to any cluster are considered as outliers. DBSCAN is able to cluster points into distinct categories without setting the number of clusters.

### 7.3. Semi-Supervised Learning of the Performance Anomalies

Although unsupervised approaches allow one to tackle a massive amount of unlabelled data, they might present worse classification performance than supervised learning methods in practice due to the lack of knowledge about the application itself. In this sense, feature extraction can improve the performance of these methods to a great extent. The primary purpose of feature selection is to remove the attributes that do not cluster well which is specially useful for distance-based clustering due to the curse of dimensionality [36]. In unsupervised problems, feature selection is usually more complicated since external validation criteria (such as labels in the underlying data) are not available. Nevertheless, if we have the label of some of the data points, supervised feature extraction methods help discover subsets of features that maximize the underlying clustering tendency. As mentioned before, we benefit from labelled data in this project. Therefore, a variety of supervised criteria can be used, such as the Fisher score. The Fisher score, discussed in Section 7.1, measures the ratio of the intercluster variance to the intracluster variance on any attribute. Our proposed semi-supervised learning method selects the most discriminative features from a small set of labelled data by means of the iterative selection method of Section 7.1. In the sequel, the DBSCAN clustering algorithm is applied to group the remaining data into the sought number of classes.

**Figure 6:**
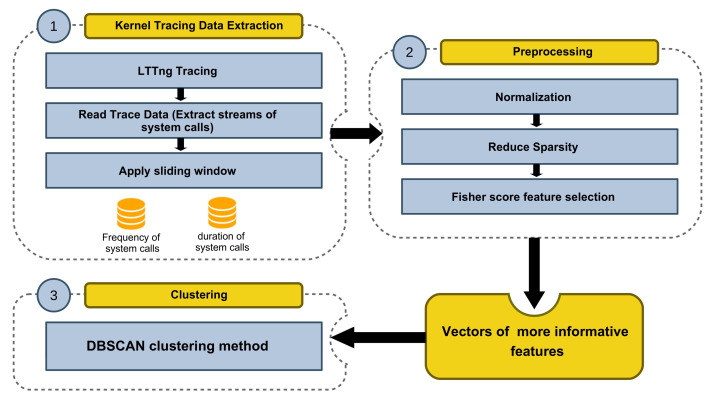
The architecture of the proposed Semi-supervised framework.

Figure 6 summarizes the proposed anomaly detection technique. The kernel tracing data extraction module, which utilized LTTng, Trace Compass, and our windowing method, has the duty of generating vectors. Then in the preprocessing module, some refinements on data are done, and the vectors of more informative features are obtained. Finally, DBSCAN clustering is applied to the obtained dataset.

## 8. Evaluation

We evaluated both proposed supervised and semi-supervised anomaly detection approaches on two real system performance anomaly datasets generated based on different faults from Mysql and Chrome applications. Our experimental setup and dataset generation is explained in Section 8.1. Then, we analyzed a practical use-case in Section 8.2. Finally, the results of the performance anomaly detection approaches are examined in Section 8.3.

### 8.1. Setup and Dataset Generation

Our experiments were performed on a group of virtual machines (VMs) allowing us to better manage system resource allocation. The host machine had an Intel Core i7 4 GHz × 8 CPU and 32 GB of memory. The VMs were equipped with different number of CPU cores and memory allocations depending on the workload simulation, running Linux Kernel version 4.15.0. As the first use case, we used the open-source MySQL synthetic benchmark tool, Sysbench 0.4.12, with OLTP test in complex mode. In order to generate the performance anomaly dataset for MySQL processes, different faults are simulated on the VMs. For example, to create a CPU issue, CPU resources allocated to a VM are limited (e.g., one CPU core, while running eight threads of MySQL). Likewise, a memory issue is created by limiting the amount of memory resources assigned to a VM (e.g., 256 MB memory, while the MySQL table is of size 6 GB). The second use case regards tracing Chrome processes. The ChromeUnderStress1.0 chrome extension is used to open, close, and refresh many light and heavy pages in Chrome with configurable speed. Faults are simulated by running this Chrome extension on the VMs with different amount of CPU and memory resources. The traces are collected using LTTng 2.10.5. The generated datasets include three classes: normal, CPU issue, and memory issue. Moreover, both MySQL and Chrome datasets are made to contain the same number of samples (i.e., 6000) from each class. We injected faults into the system for each use case using the tools we introduced. However, for other applications injecting faults is possible using two scenarios. The first scenario is injecting faults as intentional software bugs into the code. In this case, we can pause the code for *n* milliseconds and then continue, calculate π with *m* bits of precision, or other scenarios. In the second scenario of fault injection, the target is the system in which the code runs using a workload generator tool designed to subject the system to a configurable measure of CPU, memory, I/O, disk, and network stress such as Stress or Stress-ng [37] and PUMBA [38]. Besides, we must keep in mind that whether with a label or without a label, the data collection step is such that all system calls in Linux are considered. We have presented a straightforward method based on kernel tracing using LTTng, which is very light and easy to install in the system to gather all system calls information. The most informative system calls are selected in the next step, the data extraction module. Therefore, the operator does not need to know how useful each system call is, as this will be done automatically later by the framework.

### 8.2. Analysis of Practical Use-Cases

In this experiment, we analyzed the performance vulnerability due to resource Denial-of-Service (DoS) attacks. The goal of DoS attacks is to disrupt fair access to system resources. We aim to identify a class of DoS attacks in which an application consumes most of the resources so that no other useful work can be done. Thus, it maliciously destroys, for example, the memory-related performance of other applications using shared resources.

In our test scenarios, we investigate the effect of such attack on the performance of Mysql. The machine on which the Mysql is executed is made subject to attacks in few short time intervals. In order to simulate such an attack, the Stress tool has been used to keep the system’s resources in an intentionally induced state of overload or deadlock so that the system is unable to perform any other work. In another test, we simulated attacks on compression programs (zip bombs) that can involve highly recursive compressed files for which their decompression result in an uncontrolled consumption of CPU time and file descriptors. Our proposed detection scheme proves to be effective in locating the windows in which the attacks actually take place.

**Figure 7:**
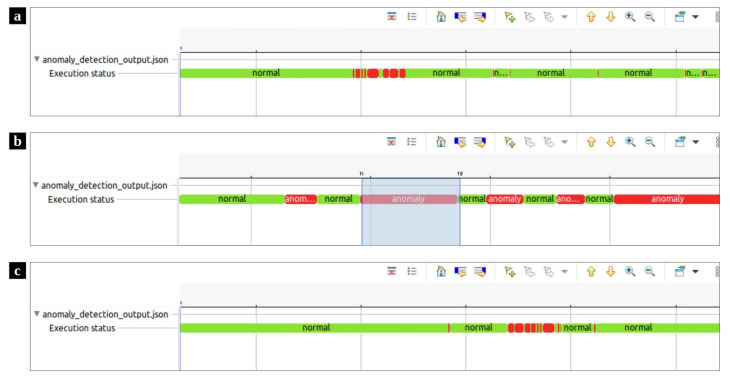
The visualized results of the test scenarios in Trace Compass time charts. (**a**) The visualized anomaly detection output where zip bombs simulated DoS attack. (**b**) the time chart provides the ability to zoom in and zoom out a specific area. (**c**) The visualized anomaly detection output where DoS attack was simulated by Stress.

Similar to the data collection phase, the trace file is read by our script in Trace Compass. Then, the whole set of system calls are formatted into windows, which are in turn analyzed by the detection module which highlights the anomalous ones. Our proposed anomaly detection framework represents the output of the detection module in a Trace Compass time chart view. Figure 7a,c demonstrate the effectiveness of our proposed method in locating the attacks that have been simulated by Stress and zip bombs. In these time charts, the normal and anomalous windows are illustrated in green and red colors, respectively. The proposed framework helps system experts to focus at just a few small windows instead of the whole trace that may include millions of events. The resulting time charts can be zoomed in and zoomed out in specific areas (Figure 7b).

**Figure 8:**
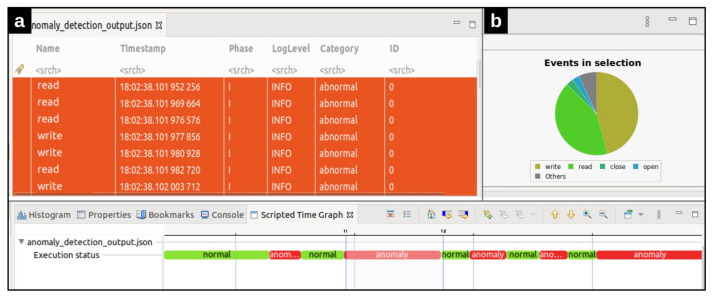
Different features our framework has offered. (**a**) The events editor table for the selected anomalous area, (**b**) The statistics chart for the selected anomalous area.

More detailed data can be computed from the trace as the user zooms in using the mouse wheel or right-clicking and dragging in the time scale. The time axis in the time chart is aligned with other views that support automatic time axis alignment. The other capability of our framework is its events editor view (Figure 8a), which presents the events in a tabular format. Filtering or searching of events in the table can be done by entering matching conditions in one or multiple columns in the header row. As can be seen in Figure 8, in addition to the original events fields, a new field has been added to each event. The field category determines whether the event belonged to a normal or abnormal window. Finally, the statistics view (Figure 8b) is provided to display the various event counters. Time synchronization is enabled between the time chart view, events editor, and statistics view. When a filter is applied in the events table, the non-matching ticks are removed from the Time Chart view (and vice versa) [25]. Moreover, the currently selected time range’s event type distribution is shown by selecting a time range in the time chart. Figure 8b shows the statistics view of the selected anomalous area detected by our tool. The distribution of events in the selected area led us to identify that the attack created by the zip bombs caused the system not to respond to Mysql requests appropriately during this period. The implementation of this visualization module which can be run using the scripting plugin in Trace Compass, is available on Github https://github.com/kohyar/syscall_anomaly_tracecompass_visualization.git (accessed on 28 July 2021).

### 8.3. Results

In this section, we evaluate the performance of the proposed anomaly detection approaches with respect to two different extracted feature spaces, one based on the duration and another based on the frequency of system calls. We deploy MySQL and Chrome processes on VMs and extract system calls from tracing the Linux kernel events to construct the feature vectors. In all experiments, the window size is $\alpha =10^{4}$ with $\beta =10^{2}$ of overlapping. At first, we conduct an experimental study on the supervised method described in Section 7.1. Then, the experimental results of the semi-supervised method are reported.

#### 8.3.1. Experimental Results of the Supervised Method

**Figure 9:**
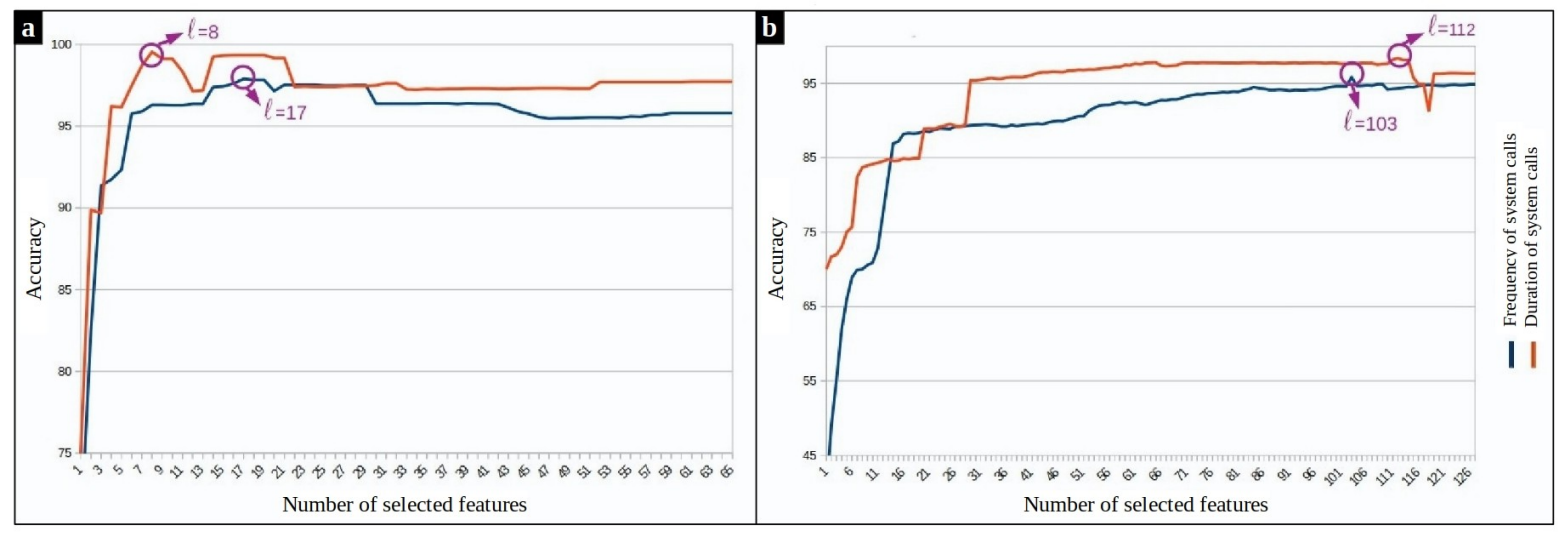
SVM-based anomaly detection accuracy versus the different number of top-ranked features. (**a**) Mysql dataset (**b**) Chrome dataset.

To tune the hyperparameters of our supervised model, 10-fold cross-validation strategy is used. One fold is used as validation and the union of other folds as training data. This process is repeated ten times for an unbiased evaluation. Fisher scores of the system calls are calculated in each run over the training set. As expected, results show that in both frequency and duration based feature spaces, some system calls have high Fisher scores, and therefore, play a more important role in separating the classes. Figure 9 shows the accuracy of the supervised anomaly detection approach during 10-fold cross-validation by varying the number *ℓ* of selected features. The experiment on MySQL processes reveals that ℓ=17 and ℓ=8 should be selected for the frequency and duration feature space, respectively. The same experiment on Chrome processes shows that the best number of features is ℓ=103 regarding frequency-based features and ℓ=112 regarding the duration-based features.

**Figure 10:**
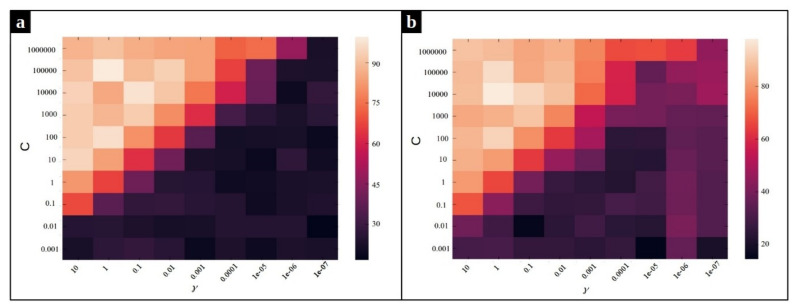
Heat map of the frequency-based and duration-based supervised anomaly detection accuracy using different parameters γ and *C* for Mysql dataset. (**a**) The heat map for frequency feature space, (**b**) The heat map for duration feature space.

**Figure 11:**
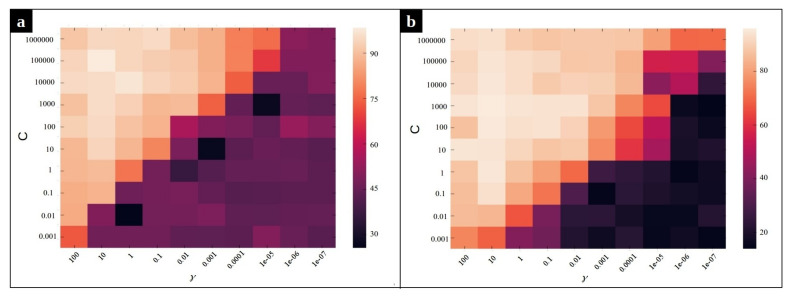
Heat map of the frequency-based and duration-based supervised anomaly detection accuracy using different parameters γ and *C* for Chrome dataset. (**a**) The heat map for frequency feature space, (**b**) The heat map for duration feature space.

As mentioned before, we employ a multi-class SVM with Radial Basis Function (RBF) kernel on our dataset to classify the input sequences into three classes: normal, CPU issue, and memory issue. The accuracy of the classification method depends on the value of two hyperparameters, *C* and γ, of the Radial Basis Function kernel SVM. Intuitively, the gamma parameter determines how far the influence of a single training example reaches, with low values meaning ‘far’ and high values meaning ‘close’. The gamma parameters can be seen as the inverse of the radius of influence of samples selected by the model as support vectors [39]. The parameter *C* is the regularization term, which controls the penalty forced on the margin for the misclassified data points. In order to optimize these hyperparameters, a grid search algorithm is performed. Figure 10 and Figure 11 depict the effect of using different combination of parameters on the average accuracy over the validation set. According to Figure 10, the pairs $\left(C=10^{4},\gamma =1\right)$ and $\left(C=10^{5},\gamma =1\right)$ yield the best SVM performance for the MySQL data set in the frequency and duration feature spaces, respectively. Likewise, we observe in Figure 11 that the pair of values $\left(C=10^{3},\gamma =10\right)$ and $\left(C=10^{5},\gamma =10\right)$ are the best for SVM on the Chrome dataset for the frequency and the duration feature spaces, respectively.

After optimizing the SVM hyperparameters, we evaluate our proposed supervised anomaly detection method on unseen test data. The accuracy, precision, and recall of the proposed RBF-SVM anomaly detection framework is reported in Table 1. These results show that both frequency and duration of system calls are useful features to perform multi-class anomaly detection, being SVM able to obtain good classification metrics by using either of them.

#### 8.3.2. Experimental Results of the Semi-Supervised Method

Following the experiment setting mentioned before, we conduct clustering experiments using K-Means algorithm and DBSCAN to evaluate the performance of unsupervised and semi-supervised performance anomaly detection. For the case of clustering, ARI (Adjusted Rand Score) is used to measure the performance [40]. ARI computes a similarity measure between two clustering solutions by considering all pairs of samples and counting pairs that are assigned in the same or different clusters in the predicted and true clusterings.

We study in Table 2 how the iterative feature selection method of Section 7.3 impacts the performance of K-Means. This table shows that the ARI of the K-Means clustering method for the frequency-based dataset by selecting the 17 and 103 features with the highest Fisher scores (i.e., ℓ=17 and ℓ=103) is 0.003 and 0.128 on MySQL and Chrome, respectively. On the other hand, for the duration-based dataset, using ℓ=8 leads to the ARI of 0.038 for MySQL samples, and the ARI of 0.018 is obtained for the Chrome samples by selecting ℓ=112. The values of *ℓ* used are the same of the previous section obtained with the supervised model.

**Figure 12:**
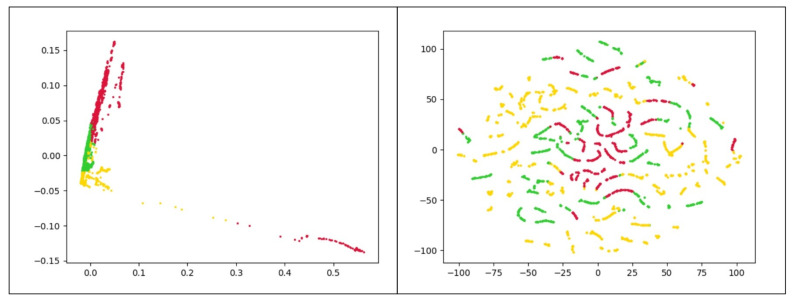
The visual result of K-Means clustering after choosing ℓ=103 features with the highest fisher score on frequency-based data set for the Chrome process; each color refers to a cluster. The **left plot** uses PCA, and the **right plot** utilizes t-SNE to map data points onto 2D subspaces.

To better explain the output of K-Means clustering, Figure 12 presents the result of this method visually. In general, these results reveal that the K-Means framework does not perform well in both duration-based and frequency-based feature spaces. This comes from the fact that the distributions of data samples in the different clusters do not have a spherical shape. In the next experiment, we analyze the performance of the DBSCAN algorithm.

The clustering results using the DBSCAN algorithm on the original feature space are shown in Table 3 for both MySQL and Chrome datasets. The parameter ϵ determines the maximum distance between two samples for one to be considered as in the neighborhood of the other. The performance of the DBSCAN method is evaluated by varying ϵ, thus obtaining different number of clusters.

**Figure 13:**
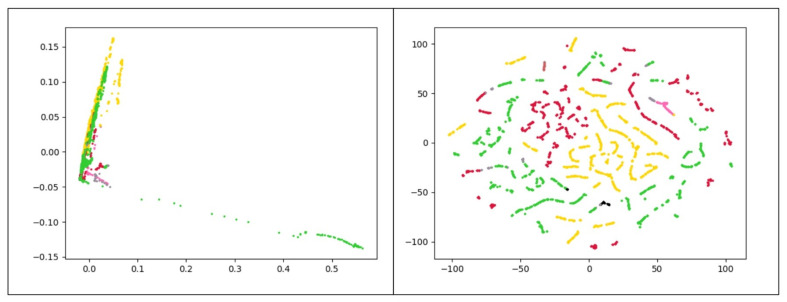
The visual result of DBSCAN clustering with $\epsilon =5\times 10^{-4}$ after choosing ℓ=103 features with the highest fisher score on frequency-based data set for the Chrome process; each color refers to a cluster. The **left plot** uses PCA, and the **right plot** utilizes t-SNE to map data points onto 2D subspaces.

The comparison results of DBSCAN are shown in Table 3. By examining the ARI using values of *ℓ* obtained in the supervised model, the DBSCAN yields an ARI of 0.874 by selecting ℓ=17 on frequency-based data set for MySQL process for which three large and five small clusters are detected. Similarly, on frequency-based data set for the Chrome process, the DBSCAN leads to an ARI of 0.823 when 103 features are selected based on the highest Fisher scores. Three large and six small clusters are obtained in this experiment. The results show that three much larger clusters are obtained in both use cases, and each of these clusters ideally contains one type of data we introduced before (normal data, memory problems, and CPU problem). The comparison of Figure 5, which shows the data points with the actual labels, and Figure 13, which illustrates the data points with labels obtained from the semi-supervised method, confirm this claim. From this table, it is clear that the performance of DBSCAN is superior to that of *K*-means. Moreover, the classification performance of DBSCAN clustering largely benefits from the supervised feature selection procedure. Finally, Table 3 displays that the proposed semi-supervised anomaly detection on the frequency-based feature space shows better ARI than the duration-based space for the mentioned processes. Interestingly, the evidence from this study intimates that by selecting the most discriminative features, the number of identified clusters by DBSCAN is decreased. This finding highlights the role of the mentioned feature selection method for mitigating the effects of the curse of dimensionality and overfitting.

To better understand the output of DBSCAN clustering model, Figure 13 displays the result of this model visually on frequency-based data set for the Chrome process. In the first plot, two principal components of PCA are used, and similarly, the second plot utilizes t-SNE [33] to map data points onto 2D subspaces.

## 9. Conclusions

In this paper, a framework for monitoring of processes and detecting performance anomalies was proposed. The framework is able to distinguish normal behavior, CPU shortage, and memory shortage in monitored traced systems. The proposed methodology works based on recording the stream of system calls using the Linux kernel tracing. From that, short sequences of system calls are extracted, and two feature vectors of duration and frequency are created to be exploited by machine learning techniques. The way we defined the data collection module makes this framework general enough to work with any specific application. Collecting system calls can be simply done on any system. Also, no special settings are used in the data collection module. Then, the extracted feature vectors are exploited by supervised, unsupervised, and semi-supervised techniques depending on the volume of available labeled data. In the supervised case, Fisher Score was applied to select the most discriminative features, and a three-class SVM algorithm was employed to detect classes. The classification performance of the method is very good, with accuracy never below 0.92. The performance of unsupervised clustering methods (i.e., *K*-means and DBSCAN) was also evaluated for the case when no prior knowledge is used. Our experiments revealed that the performance of DBSCAN is superior to that of *K*-means but not as good as that of the proposed supervised approach. Our research underlined the importance of supervised feature selection procedure (Fisher score feature selection), which is used in the proposed semi-supervised approach. Our experiments revealed that the supervised selection of features is able to boost considerably the performance of unsupervised clustering algorithms, with ARI measures as good as 0.874 regarding partition agreement. Taken together, these findings suggest that our framework is an effective tool for automated anomaly detection from traced system calls. The proposed framework along with other works done by our team will be integrated as an open-source Trace Compass extension. In the future, we will explore the performance anomalies in microservice systems using tracing data and Machine Learning. Furthermore, it would be interesting to investigate other learning models for detection of anomalies to achieve better detection performance.

## Figures and Tables

**Table 1:** The performance of the proposed supervised anomaly detection approach.

	Number of features	Accuracy	Precision	Recall
**MySQL Process **	Frequency (*ℓ* = 17)	0.928	0.989	0.968
	Duration (*ℓ* = 8)	0.937	0.988	0.978
**Chrome Process**	Frequency (*ℓ* = 103)	0.951	0.990	0.994
	Duration (*ℓ* = 112)	0.959	0.991	0.985

**Table 2:** Validation of K-Means based semi-supervised technique on original features versus where the Fisher score feature selection method is applied.

	Frequency-Based		Duration-Based	
	Data Set		Data Set	
**MySQL Process**	Original Features	0.000	Original Features	0.000
	Fisher Score (*ℓ* = 17)	0.003	Fisher Score (*ℓ* = 8)	0.038
**Chrome Process**	Original Features	0.084	Original Features	0.001
	Fisher Score (*ℓ* = 103)	0.128	Fisher Score (*ℓ* = 112)	0.018

**Table 3:** Validation of DBSCAN based semi-supervised technique on original features versus where the Fisher score feature selection method is applied.

	Frequency-Based		Number	Duration-Based		Number
	Data Set	ARI	of Clusters	Data Set	ARI	of Clusters
**MySQL Process**	Original Features ($\epsilon =10^{-3}$)	0.281	17	Original Features ($\epsilon =10^{-3}$)	0.278	18
	Fisher Score (ℓ=17 and $\epsilon =10^{-3}$)	0.874	8	Fisher Score (ℓ=8 and $\epsilon =10^{-3}$)	0.855	8
**Chrome Process**	Original Features ($\epsilon =5\times 10^{-4}$)	0.254	21	Original Features ($\epsilon =10^{-3}$)	0.127	27
	Fisher Score (ℓ=103 and $\epsilon =5\times 10^{-4}$)	0.823	9	Fisher Score (ℓ=112 and $\epsilon =10^{-3}$)	0.701	11

## Data Availability

The data presented in this study are available on request from the corresponding author.
